# A novel missense mutation of the *NAT10* gene in a juvenile Schnauzer dog with chronic respiratory tract infections

**DOI:** 10.1111/jvim.16100

**Published:** 2021-03-23

**Authors:** Barry A. Hedgespeth, Adam J. Birkenheuer, Steven G. Friedenberg, Natasha J. Olby, Kathryn M. Meurs

**Affiliations:** ^1^ Department of Clinical Sciences North Carolina State University Raleigh North Carolina USA; ^2^ Department of Veterinary Clinical Sciences University of Minnesota Saint Paul Minnesota USA; ^3^ Comparative Medicine Institute North Carolina State University Raleigh North Carolina USA

**Keywords:** ciliary dysplasia, congenital, genetics, microtubule, pneumonia, whole genome sequencing

## Abstract

An 18‐month‐old intact male Schnauzer dog was evaluated for chronic, lifelong respiratory tract infections that were unresponsive to administration of a variety of antibiotics and corticosteroids. The dog developed persistent vomiting and diarrhea around 1 year of age that was minimally responsive to diet change, antibiotics, and corticosteroids. Despite supportive care, the dog was ultimately euthanized at 20 months of age due to persistent respiratory and gastrointestinal disease. Whole genome sequencing discovered a deleterious missense A/C mutation within the *NAT10* gene, a gene essential for microtubule acetylation, appropriate ciliary development, and cytokinesis. Pipeline analysis of the genomes of 579 dogs from 55 breeds did not detect this mutation. Though never described in veterinary medicine, *NAT10* mutation occurs in humans with ciliary aplasia, suggesting a pathophysiological mechanism for this dog and highlighting an associated mutation or possible novel genetic cause of chronic respiratory infections in dogs.

**Abbreviations**
BP: base pair
DNA: deoxyribonucleic acid
NAT10: N‐acetyltransferase 10
RI: reference interval
rRNA: ribosomal ribonucleic acid
UTR: untranslated region
VQSR: variant quality score recalibration
WBC: white blood cell
WGS: whole genome sequencing

## 1 CASE DESCRIPTION

An 18‐month‐old intact male Schnauzer dog was presented to the North Carolina State University Veterinary Hospital for evaluation of persistent cough and tachypnea. He was adopted from a breeder at 2 months of age, at which time he was reported as being clinically healthy. Soon after adoption, he developed a cough and was treated with a 4‐week course of doxycycline by his primary veterinarian for presumptive canine infectious respiratory disease complex. His cough improved mildly, though it never fully resolved. By 7 months of age the dog was again presented to his primary veterinarian for persistent coughing and tachypnea. Thoracic radiographs demonstrated a diffuse bronchointerstitial pattern, and over the following months he was intermittently prescribed amoxicillin/clavulanic acid, prednisone, cefpodoxime, and chloramphenicol, all of which had a minimal effect on improving his clinical signs. At 10 months of age, a complete blood count (CBC) was performed, revealing a leukocytosis characterized by a moderate mature neutrophilia. At 12 months of age, he developed persistent vomiting and diarrhea which was treated supportively with Fortiflora, metronidazole, maropitant, and a diet change to Royal Canin Gastrointestinal Low Fat. A CBC was repeated at 13 months of age, revealing a progressive leukocytosis characterized by a marked segmented neutrophilia with a left shift and moderate monocytosis. A serum chemistry panel indicated the presence of hypoalbuminemia as well as hypocholesterolemia, decreased creatinine concentration, and mild hypoglycemia.

Due to the persistence of his cough and gastrointestinal disease despite treatment, the dog was presented to a local specialty hospital for further diagnostics. Thoracic radiographs demonstrated a diffuse alveolar pattern, and bronchoalveolar lavage cytology exhibited septic suppurative inflammation with phagocytosed intracellular bacteria. Bacterial culture of this fluid grew *Staphyloccocus*, *Streptococcus*, and *Bordetella* species. Esophagogastroduodenoscopy was performed, and pinch biopsies obtained during this procedure identified moderate lymphoplasmacytic inflammation. The dog's diet was transitioned to Royal Canin Ultamino and he was prescribed doxycycline and enrofloxacin for his bacterial bronchopneumonia. His gastrointestinal disease improved with the diet change; however, his cough and tachypnea persisted. He was therefore prescribed doxycycline, enrofloxacin, and prednisone intermittently every 3‐4 weeks to provide mild relief of his respiratory disease.

Upon presentation to our institution 6 months later, the dog was abnormally small in stature, weighing 3.7 kg and having a body condition score of 2/9. Vital signs were unremarkable aside from constant tachypnea (respiratory rate 100 breaths/minute). Mild serous nasal discharge was noted bilaterally, and occasional crackles were heard during thoracic auscultation. A CBC revealed the presence of mild regenerative anemia (hematocrit 39.0%; RI 40.2‐61.2%) (reticulocytes 139.0 × 10^3^/μL; RI 8.0‐93.7 × 10^3^/μL), thrombocytosis (547 × 10^3^/μL; RI 190‐468 × 10^3^/μL), and persistent leukocytosis (53.330 × 10^3^/μL; RI 2.841‐9.112 × 10^3^/μL) characterized by a marked segmented neutrophilia (42.664 × 10^3^/μL; RI 2.841‐9.112 × 10^3^/μL) with a left shift (band neutrophils 0.533 × 10^3^/μL; RI 0/μL), moderate monocytosis (3.733 × 10^3^/μL; RI 0.075‐0.85 × 10^3^/μL), mild lymphocytosis (5.867 × 10^3^/μL; RI 0.594‐3.305 × 10^3^/μL), and the presence of a population of atypical lymphocytes (2.667 × 10^3^/μL; RI 0/μL). A serum chemistry panel corroborated previous findings, indicating the presence of hypoglycemia (61 mg/dL; RI 75‐126 mg/dL), decreased blood urea nitrogen (8 mg/dL; RI 11‐27 mg/dL) and creatinine (0.3 mg/dL; RI 0.5‐1.4 mg/dL), hypoalbuminemia (2.4 g/dL; RI 3.2‐4.3 g/dL), hypocholesterolemia (73 mg/dL; RI 151‐348 mg/dL), and hypomagnesemia (1.4 mg/dL; RI 1.9‐2.5 mg/dL). A bile acids tolerance test was within normal limits. Flow cytometric analysis of the dog's peripheral blood confirmed that all subsets of lymphocytes were present in addition to marked granulocytosis. Despite its insensitivity for diagnosis of mucosal IgA deficiency, serum IgA was measured and found to be mildly decreased at 27 mg/dL (RI 35‐270 mg/dL). Because of the dog's chronic respiratory infections that had been present from a very young age, ciliary dyskinesia was considered a possible differential diagnosis and a semen sample was attempted to be collected to evaluate the dog's sperm motility. Sample collection was unsuccessful, and so a 2 mL blood sample was collected for genomic DNA evaluation that could aid in the diagnosis of heritable diseases including neutrophil adhesion defects, ciliary dyskinesia, or immunoglobulin deficiency. The dog was discharged with azithromycin and recommendations for continued supportive care. Despite additional courses of antibiotics, the dog's respiratory status progressed to dyspnea, and due to a poor quality of life, he was ultimately euthanized at 20 months of age with no necropsy performed.

Genomic DNA was extracted using the QIAamp DNA Blood Kit standard protocol (Qiagen, Germantown, Maryland) and submitted for whole genome sequencing (WGS). DNA was processed for library preparation and whole genome sequencing using a 150 base pair (bp) paired‐end read configuration on an Illumina HiSeq 4000 high‐throughput sequencing system (Genewiz LLC, South Plainfield, New Jersey). Variant calling from next‐generation sequencing data was performed using a standardized bioinformatics pipeline for all samples as described previously.^1^ Sequence reads were trimmed using Trimmomatic 0.32 to a minimum Phred‐scaled base quality score of 30 at the start and end of each read with a minimum read length of 70 bp, and aligned to the canFam3 reference sequence using BWA 0.7.13.^2^, ^3^ Aligned reads were prepared for analysis using Picard Tools 2.8 (http://broadinstitute.github.io/picard) and GATK 3.7 following best practices for base quality score recalibration and indel realignment (Broad Institute, Cambridge, MA).^4^, ^5^, ^6^ Variant calls were made using GATK's HaplotypeCaller walker, and variant quality score recalibration (VQSR) was performed using sites from dbSNP 146 and the Illumina 174 K CanineHD BeadChip as training resources. A VQSR tranche sensitivity cutoff of 99.9% to SNPs and 99% to indels was used for downstream analyses; genotype calls with a Phred‐scaled quality score <20 were flagged but not removed from the variant call set.

Data was filtered through a previously established database of 288 dogs of 48 different breeds (Supporting Information Table S1). Mutations were categorized by severity of potential impact using Variant Effect Predictor 91. High and moderate impact variants, including frameshifts, insertions and deletions, stop‐gained, missense, splice region, 5′ untranslated region (UTR), and 3′UTR, were evaluated based on predicted functional effects of the gene. For variants that resulted in a change to the amino acid sequence, SIFT (https://sift.bii.a-star.edu.sg/), Polyphen (http://genetics.bwh.harvard.edu/pph2/), and Provean (http://provean.jcvi.org/index.php) were used to determine if the sequence alteration was expected to significantly affect protein function.

**FIGURE 1:**
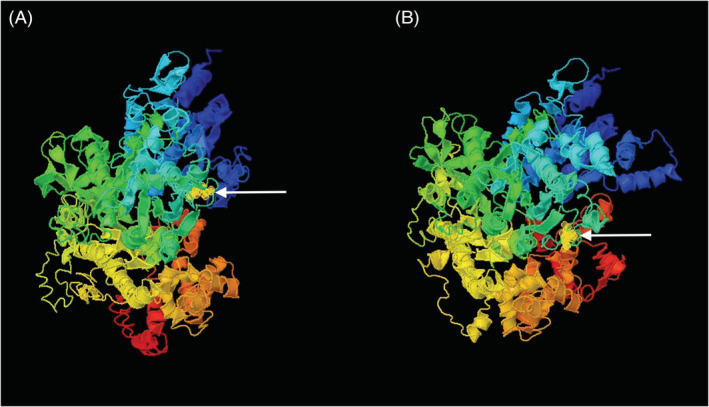
Wild‐type (A) and mutated (B) quaternary structure of the assembled NAT10 protein. Amino acid in each structure is represented by yellow circular module and annotated by white arrow

**FIGURE 2:**
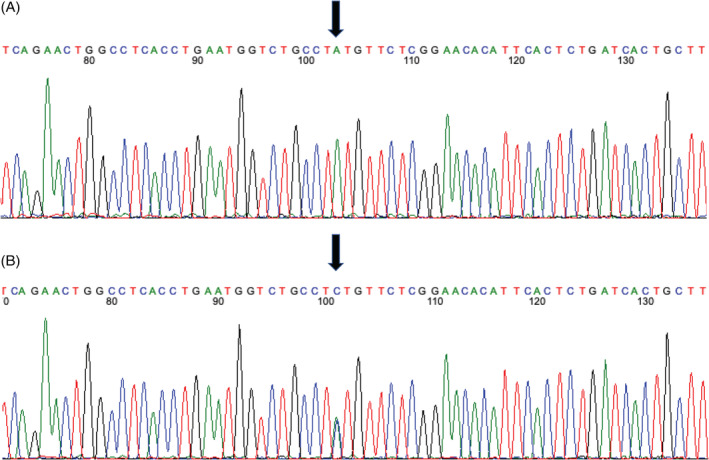
Amino acid sequence of a portion of the *NAT10* gene in an unaffected Boxer dog (A) and the mutated sequence of the dog described in this report (B), depicting a mutation from adenosine to cytosine (arrow) and subsequently changing a highly conserved positively charged histidine to a polar uncharged glutamine in the final protein structure

The analysis identified 2469 novel variants in the affected dog but only 3 of them were considered to be deleterious based on published criteria.^7^ All 3 variants were single base pair missense changes and were in the *RELN(18:16275972)*, *SNED1(25:51085013)*, and *NAT10 (18:33675758)* genes. The variants in RELN and SNED1 genes were excluded due to the lack of a clear role for these genes in the clinical presentation described. The variant in the *NAT10* gene (ENSCAFG00000007028) was a heterozygous A/C missense mutation on chromosome 18 at position 33675758 (Figure 1). The variant changed the amino acid from a highly conserved positively charged histidine to a polar uncharged glutamine (Figure 2). The variant was predicted to be a deleterious change by all 3 in silico prediction algorithms. Polyphen had a score of 0.97 (scores of 0.85‐1.0 are predicted to be deleterious), SIFT had a score of 0 (scores of 0‐0.05 are predicted to be deleterious) and Provean had a score of −7.8 (scores of −2.5 or less are predicted to be deleterious).

The variant was subsequently evaluated in a pipeline of a total of 579 dogs from 55 breeds and was not present in the genomes in any of these dogs including 37 miniature Schnauzers.

## 2 DISCUSSION

In the dog reported here a novel deleterious variant was identified in the *NAT10* gene that was predicted to create a deleterious change in the protein's structure. The NAT10 (N‐acetyltransferase 10) protein is highly expressed in all human tissues, whereas in dogs it is highly expressed in many tissues including the lungs, brain, heart, and reproductive and gastrointestinal tracts.^8^, ^9^ NAT10 is a lysine acetyltransferase that acts on microtubules and histones, and is required for 18S rRNA biogenesis, nucleolar architecture, cytokinesis, and mitosis.^10^, ^11^, ^12^ The structural change in the protein predicted here could have potentially negatively impacted the function of the protein, resulting in microtubule acetylation dysfunction. Tubule acetylation by NAT10 is critical for the development of nonmotile and motile cilia as shown in a human patient with a novel variant of *NAT10* diagnosed with heterotaxy and ciliary dysplasia; further experiments in zebrafish confirmed the requirement of NAT10 for proper left–right patterning, suggesting a vital role in embryonic ciliary development.^13^ It is possible that the dog's recurrent bacterial pneumonia was secondary to ciliary dyskinesia, aplasia, or both as a result of inappropriate NAT10 function. Depletion of NAT10 results in impaired cytokinesis and accumulation of cells in the G2/M phase.^14^ This would be especially detrimental for rapidly dividing cells such as those in the gastrointestinal tract, possibly explaining the dog's chronic gastrointestinal disease. Impaired cellular development and cytokinesis might have also contributed to the dog's small stature and the development of dysfunctional leukocytes, thus further predisposing to chronic respiratory and gastrointestinal infections. However, chronic antibiotic usage resulting in gastrointestinal dysbiosis could have also contributed to the dog's chronic gastrointestinal disease.

Although still in its relative infancy in veterinary medicine, whole genome sequencing has proven to be invaluable in expanding our comprehension of uncommon clinical presentations in animals. Animal models for human diseases such as Duchenne muscular dystrophy^15^ and neuronal ceroid lipofuscinosis^16^ have been discovered using WGS, which could aid in preclinical testing of therapeutics to ultimately benefit both humans and veterinary species with these conditions. Genomic evaluation of affected familial counterparts could provide the genetic etiology and heritability patterns of diseases, thus allowing the ability to screen future litters for such conditions as well as whether they might be amenable to novel therapeutic techniques such as gene therapy. A major limitation of this case is a lack of ciliary analysis to appropriately determine whether dysfunction or aplasia was present. Anecdotally, no other members of this dog's family exhibited similar clinical features, suggesting a de novo rather than a congenital mutation; however, further familial genetic or proteomic studies of dogs with this mutation would be required to determine the heritability of this mutation as well as its functional impact. The relative scarcity of reports of *NAT10* mutations in the human literature would suggest de novo mutation, although this could reflect the general paucity of WGS of human patients with similar presenting clinical signs. Another limitation is the lack of NAT10 expression analysis in this dog's tissues to determine the presence of aberrant NAT10 protein. Limitations of WGS such as cost and equipment availability currently preclude extensive testing of animals, but by better streamlining this process, it could 1 day be possible to utilize WGS more readily to improve our understanding of both common and uncommon disease presentations.

## 3 SUMMARY

This study has identified a novel mutation of the *NAT10* gene in a young Schnauzer dog with chronic respiratory tract infections, gastrointestinal tract abnormalities, and chronic leukocytosis. In silico analysis of this mutation predicted it to be deleterious, and despite aggressive supportive care, the dog was euthanized due to a poor quality of life. Consideration of *NAT10* mutation should be given to similar case presentations, and whole genome sequencing could aid in confirmation of this diagnosis. Further identification of this mutation in dogs might help to elucidate its heritability, any beneficial therapies for these dogs, and to possibly identify a comparable condition in humans that could be used as a reference for treatment.

## CONFLICT OF INTEREST DECLARATION

Authors declare no conflict of interest.

## OFF‐LABEL ANTIMICROBIAL DECLARATION

Authors declare no off‐label use of antimicrobials.

## INSTITUTIONAL ANIMAL CARE AND USE COMMITTEE (IACUC) OR OTHER APPROVAL DECLARATION

Authors declare no IACUC or other approval was needed.

## HUMAN ETHICS APPROVAL DECLARATION

Authors declare human ethics approval was not needed for this study.

## Supporting information


**Table S1** List of dog breeds filtered against during whole genome sequencing analysis.
