# Supplementary material for: A novel missense mutation of the NAT10 gene in a juvenile Schnauzer dog with chronic respiratory tract infections
Source: J Vet Intern Med. 2021 Mar 23;35(3):1542–6. doi: 10.1111/jvim.16100 (PMC8162599; doi:10.1111/jvim.16100)
Supplement: Supplementary file 1 — Table S1 List of dog breeds filtered against during whole genome sequencing analysis. [file JVIM-35-1542-s001.pdf]

List of dog breeds filtered against during whole genome sequencing analysis.

Breeds used for WGS filtering
American Staffordshire
American bulldog
Australian shepherd
bichon frise
border collie
boxer
Boykin spaniel
cairn terrier
cavalier King Charles
collie
corgi
dachshund
Doberman pinscher
English bulldog
English cocker spaniel
English mastiff
French bulldog
German shepherd
Golden retriever
goldendoodle
great Dane
great Pyrenees
havanese
Irish setter
labradoodle
Labrador retriever
Lhasa Apso
Maltese
miniature poodle
miniature schnauzer
mixed
Pomeranian
Portuguese water dog
pug
Rhodesian ridgeback
rottweiler
Scottish deerhound
Scottish terrier

sheltie
shih tzu
Siberian husky
standard poodle
toy poodle
Welsh springer spaniel
Welsh terrier
whippet
whwt
Yorkshire terrier
